# Lessons learned in animal acoustic cognition through comparisons with humans

**DOI:** 10.1007/s10071-022-01735-0

**Published:** 2022-12-27

**Authors:** Marisa Hoeschele, Bernhard Wagner, Dan C. Mann

**Affiliations:** 1 Acoustics Research Institute, Austrian Academy of Sciences, Wohllebengasse 12-14, 1040 Vienna, Austria; 2 Konrad Lorenz Institute of Ethology, University of Veterinary Medicine, Vienna, Savoyenstrasse 1, 1160 Vienna, Austria

**Keywords:** Music, Language, Acoustics, Communication, Vocalization, Comparative cognition

## Abstract

Humans are an interesting subject of study in comparative cognition. While humans have a lot of anecdotal and subjective knowledge about their own minds and behaviors, researchers tend not to study humans the way they study other species. Instead, comparisons between humans and other animals tend to be based on either assumptions about human behavior and cognition, or very different testing methods. Here we emphasize the importance of using insider knowledge about humans to form interesting research questions about animal cognition while simultaneously stepping back and treating humans like just another species as if one were an alien researcher. This perspective is extremely helpful to identify what aspects of cognitive processes may be interesting and relevant across the animal kingdom. Here we outline some examples of how this objective human-centric approach has helped us to move forward knowledge in several areas of animal acoustic cognition (rhythm, harmonicity, and vocal units). We describe how this approach works, what kind of benefits we obtain, and how it can be applied to other areas of animal cognition. While an objective human-centric approach is not useful when studying traits that do not occur in humans (e.g., magnetic spatial navigation), it can be extremely helpful when studying traits that are relevant to humans (e.g., communication). Overall, we hope to entice more people working in animal cognition to use a similar approach to maximize the benefits of being part of the animal kingdom while maintaining a detached and scientific perspective on the human species.

## Introduction

Early work on animal cognition focused on primates and just a few common laboratory species such as rats and pigeons. Today, it is a flourishing field with many species represented from across the animal kingdom, including species of mammals, birds, reptiles, fish, amphibians, and even invertebrates. It has now become clear that cognitive traits that were once considered to be “complex” are found in what once would have been considered unlikely species. To give just a few examples, bumblebees socially learn (*Bombus terrestris;* Alem et al. 2016) and have same/different concept learning abilities (*Bombus impatiens;* Brown and Sayde 2013), fish show transitive inference (i.e., if *A* > *B* and *B* > *C* then they infer that *A* > *C*; *Astatotilapia burtoni*; Grosenick et al. 2007), and several bird species have been attributed with mental capacities long thought to be limited to primates (Güntürkün and Bugnyar 2016).

However, there is one animal where researchers have a tendency to take cognition for granted, and that is our own species: humans. Because of direct day-to-day human experiences, researchers inadvertently collect a lot of insider information that is not available for other species. Due to this insider perspective, human behaviors are often considered to be both well-understood and too unique to be comparable to other species. For example, it is generally not expected that other animals will build something as complex as spaceships: a complex tool made up of many simple tools. This may be the reason that researchers often do not consider humans when discussing animal tool use. Similarly, it is generally not expected that animals have a complex communication system akin to language, so humans are often left out of the equation when studying animal vocal communication. There are several problems with how the human species is treated in animal cognition research.

For one, the day-to-day experience all researchers have with the human species cannot be taken as scientifically valid. While this experience is critical for generating hypotheses, this day-to-day experience is inherently anecdotal, and leaves understanding on a different level than is typical in animal cognition experiments. The average human, for example, knows what they like, but they do not necessarily know what evolutionary benefit this taste might be based on. Most of the time humans are probably unable to answer any of Tinbergen’s (1963) four questions about behavior they themselves exhibit. For example, imagine asking a person who is dancing what the adaptive purpose of dance is, how it evolved, what mechanisms underlie dancing, and when during development they first started to dance. The person would likely not have an answer for at least the first three questions and be hazy in their response to the last (e.g., “as long as I remember”). In contrast, when studying other species, the focus is on answering these questions, while little is known about the actual animal experience, and reasons they might give for doing something (e.g., “it feels good”). While these experiential reasons for doing something might lead to testable hypotheses (e.g., “Is rhythmic movement associated with hormonal release and that’s why it feels good?”), often it is just taken for granted that this is something humans do without investigation into the “why”. Also, because of this lack of insight into other species, it is common to downplay the behavior of non-human animals relative to human behavior.

Even when humans are studied, there is a tendency to study them using different methodology compared to other species. Sometimes this different testing style can lead to very different results. For example, until recently, humans appeared to be unique in how they dealt with risky decision making. In the classic research on risky decision making, humans were given surveys asking them how they would respond in hypothetical situations. In a hypothetical win situation where participants were asked if they wanted to win $100 or have a 50% chance to win $200, they were risk averse and chose $100. However, when asked if they wanted to lose $100 or have a 50% chance of losing $200, participants chose the risky option (Kahneman and Tversky 1979). Pigeons (*Columba livia*) that were trained to make the same decisions in an operant task did exactly the opposite: they took a risk if they were presented with a potential gain, and they were risk averse in the loss situation. However, decades after the initial human research, humans that were tested in the same operant paradigm as the pigeons behaved in the opposite way from the initial human studies: they behaved exactly like pigeons (Ludvig et al. 2014). This was surprising, because the idea that humans are risk averse in gain situations and risk seeking in loss situations was accepted as common knowledge in behavioral economics, whereas studying humans in the same way that one would study another species shows the opposite conclusion (Ludvig and Spetch 2011)! In other words, studying humans from an animal cognition perspective forced researchers to reevaluate what was accepted as fact about the human species.

Secondly, not only are studies with humans often conducted differently than other species, critically how the problem would even be approached from the outside is often not even considered—i.e., approaching the problem as if researchers were not themselves humans. Instead, studies are often based on an anecdotal opinion of the human species without considering where this knowledge came from and what it would take to be able to achieve similar knowledge about humans from the outside. For example, sometimes when confronted with a problem and uncertainty as to how to solve it, humans suddenly experience a “eureka” moment where the solution of the problem seems to suddenly come to mind. This phenomenon, in contrast to an incremental form of problem solving, is referred to as “insight” and, for nearly 100 years, researchers (such as Köhler and Duncker) tried to study whether other animals had it with the assumption that insight was a “complex” ability that was not rooted in “simple” processes such as associative learning (Osuna-Mascaró and Auersperg 2021). Yet this appears to have been based entirely on the human subjective experience of insight. Indeed, the difference between insight and analytical reasoning seems to depend purely on the inner experience. Now, with new methods in neuroscience it may be possible to understand what the inner experience of other animals is like. But, as Osuna-Mascaró and Auersperg (2021) argue in their review, the fact that the human experience is rewarding and feels intelligent is not a sufficient reason to label it as a “complex” feat. Insight along with many other experiential phenomena such as consciousness or intentionality or even (depending on the specific context) abstract thinking are difficult enough to demonstrate in other humans. Depending too much on a subjective experience leads to identifying constructions that may be difficult or impossible to identify in other animals.

**Fig. 1:**
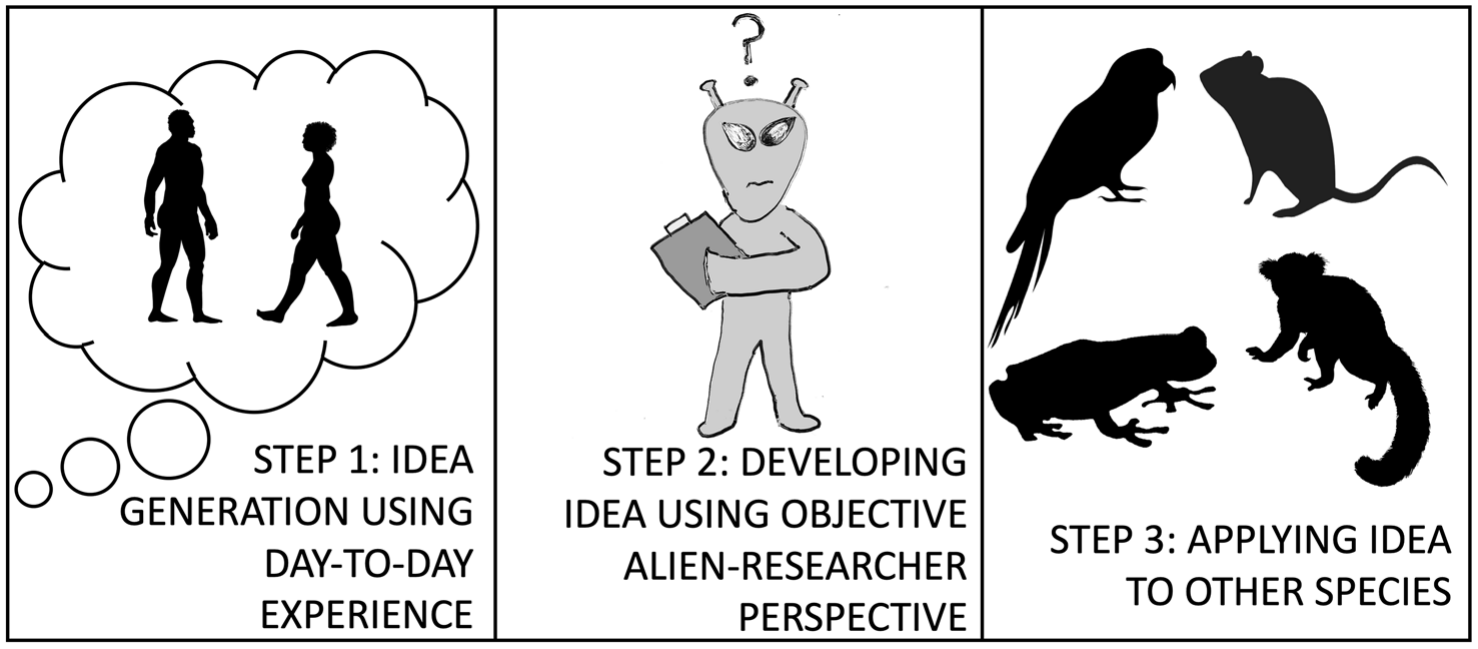
The three-step process of the objective human-centric approach to animal cognition

To be an appropriate comparative researcher, one needs to include humans just like any other species in studies of animal cognition. Insider information can be handy at coming up with potential research ideas, but before taking this information as fact it is important to take a step back and view humans as just another species. The best way to do this after coming up with an initial idea is to channel the idea of an alien researcher, no more familiar with the human species than humans are with other animals, and attempting to formulate observations and derived hypotheses from this perspective. We refer to this process as an objective human-centric approach to animal cognition. This process is a three-step process: (1) identify interesting research questions via human-centric knowledge (2) take a step back and treat the human species objectively, like any other species, and finally (3) applying these objective findings to other species. This process is displayed in Fig. 1.

Our specific subarea of research concerns the acoustic cognition of animals. Below we provide some examples of how this objective human-centric strategy has changed the way we approach animal cognition. These examples serve to both highlight important research findings in the acoustic cognition of animals and also to serve as inspiration for how incorporating this approach can enhance the study of animal cognition also in other subareas of study. But first, we start with a summary of how to study animal cognition with an objective human-centric approach.

Critically, we are not advocating for all of animal cognition research to make use of this approach. Indeed, in the past researchers made exactly this mistake: focusing too much of comparative effort on human-centric abilities and ignoring many other facets of behavior across the animal kingdom. We believe that because of this early overly anthropomorphic approach (and many of the issues that can arise with human comparisons that we reviewed above), the perceived usefulness of including humans in animal cognition has diminished. Instead of replacing current practices in animal cognition, we present the objective human-centric approach as an additional tool and a way to successfully reintegrate the study of humans into animal cognition.

## Using humans to approach the study of animal cognition

The three-step process in Fig. 1 is in many ways easier said than done. Applying an alien-researcher perspective on informal observations of human behavior and cognition has to take into account several important points. Here we outline some of the critical factors to consider when implementing this approach. Afterwards, we will delve into specific examples and then conclude with a more detailed step-by-step flowchart of how to implement this approach more broadly in animal cognition.

### Which animals to study

In the past, a first approach in animal cognition has often been to study animals close at hand. These tended to be common model-species animals such as primates, pigeons and rats. Over time, however, the value of studying a wider array of species has gained ground. These originally studied, more common species can be very useful, however, for studies of phenomena that one might expect to be commonly found in the animal kingdom, or if they happen to exemplify a species that is more directly relevant for study as we explain below. If they do happen to fit a species criterion, these heavily studied species can be useful because there are many previous studies to provide a foundation for the implementation of further studies. But how does one decide on species criteria?

Because our approach begins with humans, one obvious species group to consider is other primates. If one observes a significant difference between humans and other primates one can consider what particular human adaptations are relevant for a specific phenomenon. Conversely, if one finds similarities between humans and other primates with regards to a specific phenomenon, one can widen the search to e.g., mammals, or vertebrates to see how widespread this phenomenon is and when, in human evolutionary history, it likely arose. This is an approach based on *homology*.

Another approach is to identify species that share some relevant traits with humans based on hypotheses about why the phenomenon of interest arose. For example, if one hypothesis is that the phenomenon arose because of humans living in large social groups one might study other animals with large social groups and compare them to those that do not. In the best case scenario, two closely related species where one lives in a large social group and one does not would make ideal test subjects. Or one might study other animals that vocally learn, or other animals that use tools. Essentially, one can test whether other species that share certain traits necessarily also will display the phenomenon of interest. This is an approach based on *analogy*.

### What and how to study

We have now addressed which animals can be chosen to study. But what topics should one study in an objective human-centric approach to animal cognition? In the past, we noticed that research often took any phenomenon known to occur in humans, without direct empirical study, and tested whether another species may display behavior that suggests a similar phenomenon. In the following section (“Human cross-cultural universals in action” section) we will review many specific examples of this approach and how we can successfully move away from it. As we have outlined in the introduction, there are many problems with this naive approach. Direct empirical study in the same manner in humans as other species is critical to be able to draw any comparative conclusions. In addition, a phenomenon that may seem like a common human phenomenon based on one’s life experience may turn out to be very culturally specific and not inherently part of what it means to be human. Thus, designing studies for direct empirical study requires taking insider knowledge about humans with a grain of salt by (1) considering cross-cultural similarities and differences, and (2) identifying how one might view the same phenomenon if it occurred in another species.

A first consideration is the cross-cultural significance of a trait of interest. In some cases, a phenomenon commonly found in day-to-day human experiences is simply a culturally specific phenomenon. For example, a particular hairstyle, such as the hipster bun, may be common at a specific time in some regions. It would not make sense to compare hipster buns to the grooming practices of other species. However, the more general phenomenon, that humans value hair presentation, may be relevant for cross-species analysis. The example we gave here may seem obvious, but this is not always the case. Many phenomena one may assume are cross-culturally valid may actually be specific to, e.g., WEIRD (Western, educated, industrialized, rich, and democratic) populations. Henrich et al. (2010) coined this term in a review where they argue that researchers too quickly will assume wide ranging results, in fields ranging from decision making to visual illusions, are universally true of humans when they are not. There are many examples of this even early in human development: human children have much more variability in how much they cry or how soon they walk than was initially believed in the Western scientific community (Amir and McAuliffe 2020). Considering cross-cultural variability is therefore of utmost importance when deciding on the scope of the trait that should be considered. We believe that, rather than compare a culturally specific trait (e.g., the Müller-Lyer visual illusion, Segall et al. 1968), comparing the general phenomenon (e.g., humans can be affected by visual illusions under certain circumstances) makes more sense. Importantly, while the trait should be something that is commonly represented cross-culturally, it does not need to be common within-cultures to be studiable across species. For example, the impact of disabilities or deficits on cognitive phenomena, or the correlation between environments and antisocial behavior are both areas of research that are generally relevant to humans cross-culturally without affecting all individuals. Both could benefit from cross-species study, for example, by creating animal models of these phenomena to determine how they arise. In sum, it is important to identify the trait of choice at a level that takes into account cross-cultural differences.

Then, after identification of a cross-culturally important trait and before moving to studying animals, a critical second step is to demonstrate this phenomenon in humans as one would in any other species. For example, one could accomplish this using an experience-based operant conditioning paradigm much like in the risky choice example described above. In fact, any behavioral paradigm that does not involve asking participants how they feel or think about something can theoretically be applied to other animals. However, this information should be added at the end of an experiment where possible to provide additional information—at the end is best so as not to prime participants. Note however, that the application is not necessarily trivial: it is important to consider sensory/perceptual differences as well as motivational differences/differences in ecological relevance between species. For example, the stimulus set may need to be adjusted either by degree (e.g., increasing the frequency of sounds to make them discriminable to a species with a higher frequency range of best hearing) or even by kind (e.g., using olfactory stimuli instead of visual stimuli for an animal with limited visual but more well developed olfactory abilities). Additionally, the nature and/or rewards of the task may also need to be adjusted based on species-specific needs. For example, for humans monetary reward is usually used, in other species food rewards are often used, but other rewards (e.g., such as mate access, shelter access, access to warmth etc.) may be more fruitful both based on species (e.g., for animals that need to eat rarely such as snakes) and based on the nature of the task (e.g., if the ability is not expected to be used during food seeking, e.g., Shettleworth 1975). If one already has a species in mind, developing a paradigm in humans that can be most easily applied to that species is a highly useful endeavor.

To make our approach less abstract, below we outline some examples in our research area: acoustic animal cognition. In the final section, we draw conclusions based on the successes of this approach and provide a flowchart on how to apply this approach to other subareas of animal cognition.

## Human cross-cultural universals in action

In the following three subsections we discuss three human phenomena that might seem quite exceptional/odd to an alien researcher. The first is rhythm in music: the fact that humans move together in time using acoustic signals. The second is the use of the harmonic series in music: a fascinating cross-cultural use of the physics of sound. Finally, we will talk about the vocal units that make up human language and whether they are truly unique.

### Rhythm

Rhythm plays a very important role in music. Humans use rhythm to be able to cooperatively engage in musical activities: musicians use the rhythm to synchronize their sounds to each other. People listening to the music may use the rhythm to dance in time with the music and each other. It allows a crowd at a concert to move together and creates a feeling of togetherness.

Early research in rhythm in other species generally focused on avian species. The reason for this was mainly based on the anecdotal idea that birds create something akin to “music” because they sing what is referred to as “songs”. Some music theorists were already claiming that different bird species sang rhythmically over a hundred years ago (e.g., Billroth 1895, pp. 23–24). This initial intuition ended up being supported by later empirical work. Songbirds have strong neural and behavioral parallels to human vocal learning (Jarvis 2007) that are not found in other primates. Songbirds are true vocal learners in the classical sense (by “classic” we mean that these birds learn entirely new vocalizations based on experience), whereas non-human primates, at best, have only more subtle forms of vocal learning (e.g., modification of unlearned vocalizations; Tyack 2020).

Early studies with songbirds showed that these birds can perceive rhythmic information in sound signals. Reinert (1965) showed that jackdaws (*Coloeus monedula*) were able to distinguish a strong–weak-strong–weak pattern from a strong–weak-weak pattern at different tempos and timbres. Hulse et al. (1984) showed that European starlings (*Sturnus vulgaris*) could distinguish rhythmic from arrhythmic patterns where the rhythmic patterns had standardized tone lengths and inter-tone-intervals while both tone lengths and inter-tone-intervals varied for the arrhythmic stimuli. Birds also appeared to attend to rhythmic information in their own vocalizations (e.g., Slabbekoorn and ten Cate 1999).

While these studies were promising, more recent studies with pigeons, zebra finches (*Taeniopygia guttata*), and budgerigars (*Melopsittacus undulatus*) suggested that often the birds are paying attention to what are known as “absolute” rather than “global” features to solve these tasks (Hagmann and Cook 2010; van der Aa et al. 2015; ten Cate et al. 2016). In other words, they attend to the particular lengths in a signal (absolute features) and not the abstract structure (global features) that they produce. For example, they may listen for a tone with a particular length, followed by a pause of a particular length rather than equally spaced (known as isochronous) tones. There were some exceptions though: some of the budgerigars did appear to attend to global features (ten Cate et al. 2016).

It’s quite possible that absolute rather than global features may control the perception of rhythmic features in birds’ own songs and the older studies on rhythmic perception in birds as well. This raises the question: What is really meant by musical rhythm? Something that was not considered early on is that it is exactly this abstract global structure that makes rhythm useful in human music. Because, in fact, if one considers rhythm broadly, almost all animals produce and respond to rhythms in one way or another. Many animals walk at a regular pace, breathe at a regular rate, and may produce rhythms accidentally by simply producing something at a maximal rate, such as a woodpecker pecking a tree (Wang et al. 2011), or a songbird performing a trill (Podos 1997).

Around the same time as some of the above more recent studies in animal rhythmic behavior, Patel (2006) developed the vocal learning and synchronization hypothesis. This hypothesis distinguished the kind of rhythm necessary for music from the many other forms of rhythm one might find in the animal kingdom. Specifically, humans synchronize to a “beat” in music, which can easily be identified even though the raw acoustic signal in many cases does not clearly indicate where the beat is. In other words, the beat in music is an abstraction from the audio signal. Secondly, humans can adjust their movements to a wide range of beats. And finally, humans move body parts to the beat that are not moved to create the beat itself (e.g., nodding our heads see Patel 2021). This hypothesis predicted that beat perception and synchronization depends on the ability to be able to vocally learn because of the direct auditory-motor neural connections found in classic vocal learning species and not in other species (Doupe and Kuhl 1999). Initial findings supported this hypothesis. Based on YouTube videos (Schachner et al. 2009) and experiments with a pet cockatoo (Patel et al. 2009), it seemed that animals that were able to imitate sounds were the only ones that moved spontaneously with accurate timing to human music. However, laboratory studies later confirmed that at least one vocal non-learning species, the California sea lion (*Zalophus californianus*) could be trained to track the beat in music (Cook et al. 2013).

Not only that, but many aspects of beat perception and synchronization can be found with high accuracy in other species. Some insect and frog species, despite not matching all of the criteria outlined by Patel (2021), exceed the synchronization abilities of humans (see Greenfield 2005; Jacoby et al. 2021). Is this because they are using an entirely different mechanism? Or are they a highly relevant precursor to the more abstract rhythmic behaviors found in humans? Additionally, research to date has mainly been focused on acoustic rhythms, but rhythmic behaviors do not necessarily directly create sounds. Dancing, for example, is a response to sound, but it does not create sounds. Intuitively one might assume that the sound had to have come first, but studying other primate species suggests that it may be the other way around. For example, chimpanzees form what is known as a conga line and dance together very accurately without any acoustic stimulus (Lameira et al. 2019).

Now researchers are finally at a position to begin to ask important questions about where rhythm in music actually comes from. Several critical stages were traversed in past research: in the first phase, rhythm was not defined, and other species were tested without a strong theoretical grounding for what should be looked for. In the second phase, a hypothesis was put forward defining what makes musical rhythm and where it might come from and observations were made about animals performing rhythms in their natural environments. Now it is possible to move forward with phase 3 where the components of rhythmic behavior can be identified in humans, tested using comparative methods in humans, and then similar methods applied across species. For example, one study created a place preference paradigm in humans where it could be shown that humans prefer to spend time with rhythmic (containing a steady “beat”) sounds compared to scrambled versions of the same sounds (Hoeschele and Bowling 2016). This experimental display of human preferences was important: while it seems intuitive that humans would prefer rhythmic to scrambled patterns, it is important not to make this assumption before testing animals. The same study then included a comparison with budgerigars, a species that has been shown to be able to synchronize with a beat (Hasagawa et al. 2011) to address whether rhythm is something esthetically pleasing. The study found that female budgerigars spent more time with rhythmic sounds, whereas the males did not. Because male budgerigars perform songs with headbobs for female budgerigars (Brockway 1964) this species might be ideal to study the attraction (female) and production (male) of rhythms separately (Hoeschele and Bowling 2016).

Overall, by identifying a critical, cross-cultural, and unusual ability in humans to dance and sing and move together in time an area of research has opened up that is relevant to a wide variety of species across the animal kingdom: everything from birds and mammals to frogs and insects appear highly relevant to solve the mystery of musical rhythm. Starting from human musical rhythm has placed attention on specific abilities, such as motor entrainment, that has led to interesting cross-species hypotheses about rhythm. In our view, if research had started from the bottom, e.g., defining rhythm in a broader way instead of narrowing in on musical rhythm, nuances in rhythmic behaviors might have been missed that play an important role in social interaction across species. A true alien researcher with no insider information about human rhythm may not even identify motor entrainment or disambiguate between absolute vs. global features in perception, but may be satisfied with a simple discovery that many species do attend to timing information in acoustic signals. An insider view of what drives humans is also a strong motivational force to delve deeper into the meaning of rhythmic behavior. Starting from humans has enabled researchers to identify a multifaceted suite of behaviors that may present themselves differently across species. From here, there is no reason to restrict oneself to a human perspective. Perhaps now it is possible to also find other components of rhythmicity that humans lack. In sum, humans are likely like any other species in that they have a subset of all possible rhythmic behaviors, but starting with and focusing on one species has allowed us a nuanced impression of the range of abilities that may be at play here. Humans are simply a convenient focus species because researchers have so much insider information about them.

### Consonance and octave equivalence

The next example we wish to discuss is musical phenomena that derive from the harmonic series, namely “consonance” and “octave equivalence”. “Consonance” describes the perception that simultaneously occurring notes separated by small integer frequency ratios sound pleasant (see e.g., Bowling and Purves 2015; Krumhansl 1990, pp. 3; Terhardt 1984). A musical interval known as the octave (ratio of 2:1) is the most consonant interval. “Octave equivalence” describes the perception that notes separated by an octave (i.e., a doubling in frequency) sound similar to humans (Burns 1999, pp. 215–264; Patel 2003; Hoeschele et al. 2012; Wagner et al. 2022). While there are cross-cultural differences in musical scales and the way pitch is perceived (Hove et al. 2009) the octave is a basis of pitch perception in multiple musical cultures around the globe (e.g., Burns 1999, pp. 215–264, but see Jacoby et al. 2019; McDermott et al. 2016).

The occurrence in pre-verbal infanthood of preference for consonance (Masataka 2006; Perani et al. 2010; Schellenberg and Trehub 1996; Trainor et al. 2002; Trainor and Heinmiller 1998; Trehub 2003; Zentner and Kagan 1996; 1998 but see Platinga and Trehub 2014) as well as perception of octave equivalence (Demany and Armand 1984) suggest a potential biological basis of these phenomena. Therefore it may be unsurprising that there is research in non-human animals investigating the possibility of such a biological basis.

As in previous examples we have provided in this paper, early cross-species studies began simply by naively testing whether other animals share perception of consonance and octave equivalence with humans. Some studies only tested whether animals could discriminate consonance and dissonance (dissonant sounds are the opposite of consonant sounds and usually have relatively complex frequency ratios) without testing for *preference* to consonance (Hulse et al. 1995; Izumi 2000; Watanabe et al. 2005; see also Porter and Neuringer 1984; Brooks and Cook 2010; see Toro and Crespo 2017 for a review). Some studies went further, studying whether test species actually preferred listening to consonant over dissonant stimuli. These consonance preference studies found no preference for consonance in several species (Akre et al. 2014; Koda et al. 2013; McDermott and Hauser 2004). One study finding consonance preference in an infant chimpanzee (Sugimoto et al. 2010) was later criticized for lack of control (Chiandetti and Vallortigara 2011). Another study finding consonance preference in albino rats (*Rattus norvegicus;* Fannin and Braud 1971) was later contradicted by findings from Crespo-Bojorque and Toro (2015). Because of this, the best evidence for consonance preference in non-human animals comes from field studies with birds whose songs contain sequences of consonant intervals (Doolittle and Brumm 2012; Doolittle et al. 2014; Richner 2016).

At this point, to move forward using the objective human-centric approach, required a step back, to consider the mechanisms and evolutionary importance of the studied phenomena in humans as one would do in any other species. One suggestion was that the physical structure of the human voice could be the biological basis of these phenomena (e.g., Bowling and Purves 2015; Schwartz et al. 2003; Terhardt 1984). The human voice is a harmonic sound, i.e., it is a sound where, above the fundamental frequency, overtones occur at integer multiple frequencies of the fundamental. Thus, the human voice prominently features the octave and other intervals that are considered to be highly consonant (the perfect fifth, 3:2 and the perfect fourth, 4:3). In fact, human preference for consonance over dissonance correlates with a preference for harmonic sounds such as the human voice (Bowling and Purves 2015; Bowling et al. 2017; 2018; Cousineau et al. 2012; McDermott et al. 2010). Thus, preference for the human voice could lead to preference for consonance. This hypothesis is known as the “vocal similarity hypothesis”. Most non-human animals also produce harmonic sounds, a preference for which may similarly translate into a preference for consonance. Evolutionary benefits of such a preference could be in distinguishing inanimate objects (which usually produce inharmonic sounds—see e.g., Chiandetti and Vallortigara 2011) from animate ones.

The vocal similarity hypothesis can be tested directly in cross-species studies. Will animals prefer consonance over dissonance in a situation where they are seeking a conspecific voice? In an empirical study, newly hatched chicks (*Gallus domesticus*) that had been incubated in acoustic isolation were shown to prefer approaching one of two identical imprinting objects if it was associated with a consonant as opposed to a dissonant piano melody (Chiandetti and Vallortigara 2011). Because a mother hen’s call—just like the human voice—is a harmonic sound, the chicks may have used consonance as a cue as to which object was more likely to be their mother (Chiandetti and Vallortigara 2011). Thus, this study provides an example of a species being attracted to consonance as a proxy for vocal output. Note that in this case, however, it remained unclear whether humans would prefer consonance in a similar setting. To complete the steps of our objective human-centric approach, Wagner et al. (2020) ran a comparable place preference study in humans and found that humans did spend more time with consonance than with dissonance. Note that while this validates the results from Chiandetti and Vallortigara (2011) with regards to our approach, it is where possible perhaps advisable to test humans first with a paradigm that can then be applied to non-human animals as described above. This paradigm that was verified in humans was then applied to further cross-species study by Wagner, Bowling, and Hoeschele in a parallel study of budgerigars (Wagner et al. 2020) which showed no general preference for consonance in a place preference paradigm. This result makes sense from the perspective of the vocal similarity hypothesis because budgerigar vocalizations have relatively obscured harmonic information when compared to e.g., humans (Lavenex 1999; Tu et al. 2011).

The vocal similarity hypothesis can also be seen to explain octave equivalence—after all, in any harmonic sound the fundamental frequency is presented together with its octave. However, another hypothesis posits that while harmonicity is important, vocal learning is key to perception of octave equivalence. This “vocal learning hypothesis” is derived from the empirical observation that, during vocal learning, human children will produce imitations of sounds presented by voices that are outside of the child’s vocal range by transposing the template sound’s fundamental frequency by an octave (Peter et al. 2008; 2009; see also Peter et al. 2015). An octave transposed sound shares the maximum overlap in harmonics with the template sound that any sound with a different fundamental frequency can have and is perceived by humans as an accurate imitation (see Hoeschele 2017). Therefore, vocal learning animals may have developed octave equivalence for the purpose of producing successful imitations.

Once again, cross-species studies can help here. An initial study in rats by Blackwell and Schlosberg (1943) is inconclusive as it was (much) later criticized for not controlling for harmonics which could mean that the octave was contained in a training stimulus (see Burns 1999, pp. 215–264). A second study searched for octave equivalence in a songbird species: the European Starling (*Sturnus vulgaris*; Cynx 1993). Similarly to the early studies on rhythm, a songbird species was chosen because they were thought to be an especially “musical” species. This study did not find octave equivalence in the starlings. However, problems with this study were also identified later: it turns out that, just like in the risky choice example we discussed in the introduction (Ludvig et al. 2014), the comparison with humans was not justified. This study used a paradigm that later was shown to also not be able to demonstrate octave equivalence in humans (Hoeschele et al. 2012).

Instead, using the objective human-centric approach outlined here, a nonverbal operant conditioning paradigm was created that successfully demonstrated octave equivalence in humans (Hoeschele et al. 2012). This could then be applied to a songbird species. Black-capped chickadees (*Poecile atricapillus*; Hoeschele et al. 2013) were chosen for this experiment. However, unlike the risky choice example, the conclusions did not change with a more rigorous experimental procedure: this songbird species still showed no evidence of octave equivalence.

While this study demonstrated that vocal learning was not itself sufficient for octave equivalence, the next thought was that perhaps a species that imitates sounds outside of its vocal range, just like human children (and unlike the black-capped chickadee), might be a better candidate. We therefore tested this hypothesis on budgerigars, a small parrot species that can imitate sounds outside of its vocal range such as human voices. However, budgerigars also did not show evidence of octave equivalence in a study using the paradigm developed via our objective human-centered approach (Wagner et al. 2019). Instead, the budgerigars showed an opposite effect, much like the black-capped chickadees. Therefore, vocal learning does not appear to be the deciding factor for species to perceive octave equivalence.

This conclusion that vocal learning is not the key ingredient needed for octave equivalence fits with the few studies that demonstrate octave equivalence in non-human species. Wright et al. (2000) demonstrated octave equivalence in a study with two rhesus macaques (*Macaca mulatta*), Richards et al. (1984) documented spontaneous octave transposition in an individual bottlenose dolphin (*Tursiops truncatus*), and Mann et al. (2020) documented octave imitation behavior in house finches that were tutored by canaries (though this was not the intended focus of this study). Note that even if one takes all three of these results at face value, one out of these three species is not a vocal learning species (rhesus macaques), which further supports the idea that vocal learning is not the critical component of octave equivalence.

As we assume they have a common biological basis, taking the results of cross-species octave equivalence and consonance research together allows one to reconsider why these phenomena may have arisen in humans in the first place. For example, so far, cross-species results suggest that vocal learning is not enough to cause a species to perceive/use octave equivalence and consonance, whereas harmonic clarity in species-specific vocalizations may be critical. In a recent review, Wagner and Hoeschele (2022) suggested that octave equivalence and consonance may be constrained by a more complex interplay of factors and propose four traits that appear to constrain the phenomena in humans. These four traits are: (1) vocal learning, (2) clear harmonic vocalizations, (3) differing vocal ranges, (4) simultaneous vocalization/duetting. The first two traits fit with the already discussed vocal learning and vocal similarity hypotheses. Differing vocal ranges we touched upon: in order for the harmonic series to be relevant during vocal learning, an individual needs to be imitating a sound outside its vocal range. Finally, simultaneous vocalizations and/or duetting may make it more critical that the harmonic series is taken into account so that vocalizations merge together seamlessly. Interestingly, in the human literature the cross-cultural “pleasantness” of consonant intervals has been debated (Jacoby et al. 2019; Athanasopoulos et al. 2021; but see Bowling et al. 2017). It may be that “pleasantness” is an indirect way of assessing the effects of consonance in the human species. For example, McPherson et al. (2020) found that simultaneously occurring notes were more likely to be perceived as one note if they were separated by consonant rather than dissonant intervals. In other words, notes separated by consonant intervals appear to perceptually merge. This makes a lot of sense, because a lot of harmonic information is shared between notes separated by a consonant frequency interval. This merging effect would especially be true of the octave, given that the octave has the most harmonic overlap between component notes. Indeed, it has recently been suggested that the basis of octave equivalence may be perceptual merging of sounds within a harmonic structure (Demany et al. 2021). This merging effect could also, on average, lead to increased pleasantness ratings.

Because humans have all four of the proposed traits underlying octave equivalence and consonance, they may be especially likely to attend to harmonic information. But many other species have a subset of these traits. And perhaps other species will have additional traits that humans do not have which will turn out to favor attention to the harmonic series. It is now possible to use our approach to tease apart the role of these traits in octave equivalence and consonance perception. In other words, the objective human-centric approach has provided a hypothesis-driven perspective on whether the harmonic series plays a role in a given species. This has implications that reach well beyond humans and music. Our objective human-centric approach has, also here, opened up a line of research that started from humans and has now evolved into the study of a cognitive trait across species: how do species across the animal kingdom make use of the physics of sound in their cognition?

### “Complex” vocal units

Among animal communication systems, human communication is unique. An interesting quirk of human language is that the previous statement is provocative yet banal at the same time. Adhering to the strict definition (“denotation”) of the word “unique”, no one could disagree with the statement. Of course, the same could be said for practically any other species, e.g., black-capped chickadee communication. After all, ornithologists and bird watchers can easily identify a black-capped chickadee from only the species-typical call. Furthermore, black-capped chickadees respond more strongly to their species-typical song than to the vocalizations of other species or modified versions of their own calls (Charrier and Sturdy 2005). However, if we opened a paper with the statement “black-capped chickadee communication is unique” readers would definitely be expecting something more novel and interesting. These statements must be understood in their social context, they are not simple truth value propositions. The statement that “*x* is unique” usually means something more akin to “*x* is uniquely unique”. The meaning of “uniquely unique” for human communication, language in particular, is defendable. After all, humans can generate a theoretically infinite number of utterances to communicate complex abstract thoughts and, in spite of the complexity of many animal communication systems, there is no evidence that there are other generative systems that transmit the same type of communicative information. So, why would the human uniqueness claim be provocative in this case? Historically, the default assumptions for humans and non-humans have been different. For non-humans, the burden of proof is on the person claiming uniqueness or “special”-ness. But for humans, the default is reversed; humans are considered unique until there is counter-evidence. As Hauser et al. (2002, pp. 1572) stated “it is surprisingly common for a trait to be held up as uniquely human before any appropriate comparative data are available.”

For language and speech, many of these premature claims of uniqueness seemed entirely reasonable. For instance, categorical perception—the tendency for listeners to respond to gradient acoustic stimuli as if the stimuli were categorical—seemed tailor-made for spoken language (Liberman et al. 1957). Speech is rapid and information-rich but also variable and imprecise. If a listener is unable to perceive the correct sound categories then they will be unable to extract the linguistic meaning from the signal. Categorical perception facilitates spoken language perception by abstracting away from minor acoustic variation in the signal.

In the lexical domain, researchers documented numerous abilities that children seem to implement to learn words such as “fast-mapping”, “mutual exclusivity bias”, “whole object bias”, etc. (see Bloom 2001 and the discussion therein). While some argued that these abilities emerged from domain-general cognitive processes, many believed that they were specific to human language and evolved to facilitate the acquisition of the vast human lexicon. Again, there were rational arguments to support this view point. For example, consider Quine’s (1960, pp. 29–35) “gavagai” problem. If someone says “gavagai” when a brown rabbit appears there are potentially limitless hypotheses that the child could entertain related to the meaning of “gavagai”. The word could refer to a single part of the animal, the action the animal is performing, the color of the animal, how the leaves move as a result of the animal’s movement, etc. Linguistic mechanisms that narrow the range of what is possible certainly would ease the burden on the learner and would explain why children reliably, and seemingly with little effort, build their vocabulary.

These hypotheses made sense when thinking about human language, but their proponents rarely thought deeply about non-humans. To the extent that animal data were considered, their abilities were disregarded because species that were closely related to humans, like chimpanzees, could not learn to speak or learn the same number of words as humans. However, broad scale tests of “can animals learn language” are not the most fruitful approach to comparative research.

Like other complex behaviors, language is not a single trait that exists independently from other cognitive abilities. Rather, language is better understood as a network of behaviors and cognitive abilities. To use language, humans must have a sizable memory to store lexical information, precise motor control to rapidly modulate the linguistic signal, the ability to form abstract categories, social learning, etc. Through a more fine-grained approach, ample evidence emerges that animals share most, and maybe all, of the traits that build language (Fitch 2012). For the aforementioned uniquely human traits, Kuhl and Miller (1975) and Dooling et al. (1987) found categorical perception in a non-human mammal and a parrot species, respectively. So, categorical perception may not have evolved to perceive language sounds but, rather, language may have made use of a broadly shared cognitive ability. Similarly, there is mounting evidence that language-specific mechanisms are not needed to explain human lexical acquisition. Many of the proposed specialized traits have been found in non-human animals. For instance, dogs show evidence for fast mapping (Kaminski et al. 2004) and the mutual exclusivity bias (Pilley and Reid 2011). Other traits may be inadequate models for explaining behavior in both non-human and human animals. Experiments from Wilkinson et al. (2003) and Griebel and Oller (2012) suggest that the aforementioned fast mapping data can be explained by alternative mechanisms in both dogs *and* human children. From an evolutionary perspective these data are not surprising. Traits that appear to be completely novel are typically reorganized, repurposed, or modified versions of preexisting traits (like the narwhal tusk that evolved from an incisor; Moczek 2008). To build on an analogy used by Pinker (1994), the elephant trunk—like human language—is a seemingly "novel" and "special" trait. The elephant trunk is roughly two meters long and is a muscular hydrostat—which means it can bend, stiffen, and produce force without relying on skeletal support (Kier and Smith 1985). This property gives elephants the strength to move heavy objects and the dexterity to manipulate tiny objects (like removing a thorn). Elephants also use the trunk for sound production, in particular in the production of long range rumbles (Stoeger et al. 2012). None of these traits are present in the hyrax, the elephants’ nearest extant relative. While the trunk as a whole is seemingly unique, the individual traits that make the trunk special are not. Tapirs, elephant seals, and saiga antelopes have all evolved extended nasal tracts (Taylor and Reby 2010). Saiga antelope and southern elephant seals use the extended nasal passage in sound production (Galimberti et al. 2019; Volodin et al. 2014). Manatee snouts, octopus arms, and the tongues of most mammals and reptiles are muscular hydrostats (Kier and Smith 1985; Marshall et al. 1998). Like the elephant trunk, human language can be appreciated for its complexity without treating humans as separate from evolutionary theory.

There is now widespread agreement that most of the traits that make up human language are broadly shared and, for the most part, many researchers have moved away from finding what makes humans “special”. However, the linguistic domain where claims of human uniqueness are still common is syntax. Broadly speaking, syntax is how words are arranged in a sentence. To understand why there is a heavy focus on syntax in animal-human comparative research, you have to understand the field of linguistics in the twentieth century. Noam Chomsky was part of a larger wave of philosophers, scientists, and researchers who rejected Behaviorism's "blank slate" approach to explaining behavior and cognition. There must be a reason why all humans acquire language but a dog, cat, or chimpanzee exposed to the same input do not. Furthermore, humans do not seem to just learn what is in their environment, rather, they can generate novel constructions, like Chomky’s now famous nonsense sentence "colorless green ideas sleep furiously". English speakers readily accept this as an acceptable English sentence even if they had never heard it before and even if it is meaningless (Chomsky 1957, pp. 15).

Of course, human phonology (the organization of meaningless units—technically called phones in spoken language—to create meaningful units like words) is also generative. Speakers can invent new words that are readily acceptable to others in their language community. So why was syntax seen as the trait that separated human and animal communicative abilities? One reason is that syntax seems to be hierarchically organized whereas phonology is organized sequentially.

In the sentences in Table 1a, “chamber door” is the closest noun to the verb “quoth” in terms of sequential structure. But English speakers do not interpret the “chamber door” as quothing anything. This is because the closest noun in terms of hierarchical structure is “the raven”. Examples like those in Table 1 suggest that sequential organization is not sufficient to explain human syntax (Chomsky 1957, pp. 18–25). In animal vocal behavior, there are a lot of species that produce multi-unit signals (e.g., bird song, whale song). However, many researchers contend that for the animal systems that have been described, there is no evidence that hierarchical organization is required and that they can all be handled by sequential processing (Berwick et al. 2011).

**Table 1:** Example sentences demonstrating that the sequential order of words is not sufficient to understanding syntax. In these examples, the verb is not describing the noun closest to it

(a) The raven who rapped at my chamber door quoth ‘Nevermore’	
(b) The toy for the budgies with the green feathers was bought yesterday	
(c) The budgie that Alex saw chased the student	

In terms of processing, numerous studies have been done to test if animals can learn hierarchical structures. Cotton-top tamarins were unable to learn a grammatical rule that required hierarchical structure even though they were able to learn a similar grammatical rule that only required sequential organization (Fitch and Hauser 2004). However, cotton-top tamarins do not have an extensive vocal repertoire and have, at best, only marginal vocal learning abilities (Tyack 2020), so they are not the most likely candidate for hierarchical processing abilities. Also, individuals were tested on heterospecific vocalizations and the paradigm did not use explicit training. It is possible that, with training, the tamarins could have eventually learned the hierarchical grammar rule. Gentner et al. (2006) chose to test starlings, a songbird species that is much more distantly related to humans but that is a vocal learner with a large repertoire of vocalizations. The researchers trained starlings on grammars built from conspecific song units and found that the starlings were, in fact, able to learn the hierarchically structured song. Other research groups seemed to have found evidence in other songbirds (Bengalese finches, *Lonchura striata*, Abe and Watanabe 2011; Japanese tits, Suzuki et al. 2016) and in baboons (*Papio papio*, Rey et al. 2012). However, the interpretation of these results have been called into question. While some of these species clearly learned complex, abstract rules, they could have used sequential strategies to do so (van Heijningen et al. 2009). Beckers et al. (2017) assessed the stimuli for multiple animal grammar learning experiments (including those testing hierarchical structure) and found that most of the results could be explained by acoustic similarities between the training and test stimuli. Ultimately, there is no clear evidence that non-humans have hierarchical grammar (though Jiang et al. 2018 provides compelling data that rhesus macaques can learn hierarchical grammars).

But, if humans are treated as just another species, one should be cautious to over-interpret the apparent difference between humans and animals in hierarchical organization. For one, while the hierarchical organization of human syntax is pretty widely accepted, there are critiques. Just as many of the results in the animal studies can be explained by simpler sequential rules, so can many of the human examples (Christiansen and MacDonald 2009; Christiansen and Charter 2015). More importantly, hierarchical processing/organization may be a domain-general cognitive tool available to humans but one that is used only sparingly in language processing. The Late Assignment of Syntax Theory posits that initial sentence processing is based on simpler processing tools related to more general associative meaning. Most processing might use this “pseudosyntax” until and unless a construction arises which requires a more complete analysis (Townsend and Bever 2001, pp. 158–246). Furthermore, many, if not most, of the sentences that are produced do not require hierarchical organization. In fact, center-embedded structures like Table 1a–c can be handled by a sequential grammar (Frank et al. 2012). Hierarchical structure is required to deal with the idea that humans can build infinitely embedded recursive structures. In practice, however, sentences that have more than one level of embedding are quite difficult to process (Christensen and MacDonald 2009). For example, adding another level of embedding to the sentence in Table 1c becomes more difficult to parse: “The student that the budgie that Alex saw chased grabbed the net.” In fact, in some English constructions with multiple center-embedded phrases, English raters preferred "ungrammatical" versions of the sentences where parts of an embedded phrase were removed. Gillespie and Perlmutter (2011) found that errors in subject-verb agreement (e.g., like in Table 1b: "the toy … was/*the toy… were") increased as the linear distance increased, irrespective of the hierarchical distance.

Crucially, the understanding of hierarchical organization in human language syntax has come about because language scientists have access to the meaning and nuances of their own species-typical signal. For example, to know that a noun is related to a more distant verb (rather than the more linearly closer verb), one must understand the meanings of the individual words and the meaning of the entire sentence. A hypothetical alien researcher would not have access to these internal human mental representations of meaning, they could only evaluate what is actually externalized. In both sign languages and spoken languages, the externalized signal is linear. In other words, this means if human language was evaluated like animal vocalizations, hierarchical information would not be detected. Furthermore, irrespective of the language, most of the sentences that are produced do not need to be modeled as hierarchical (Karlsson 2007). If the aliens are able to run experiments, the data are still likely to be inconclusive. Many of the human artificial grammar learning experiments suffer from the same problems that Beckers and colleagues discuss with non-human artificial grammar learning experiments (Pothos 2007), so much of the human experimental data can also be explained by simpler processes.

Using alien researchers as a thought experiment is a common exercise in the language sciences. However, consider the difficulty in analyzing a complex communication system without a prior, clear understanding of function or structure. This is of utmost importance for comparative research. Human scientists are in the same predicament with respect to non-human communication. If a team of alien researchers approached human language the way that humans have to approach animal communication, they would likely fail to discover many of the traits that many have argued make human language “special”.

**Fig. 2:**
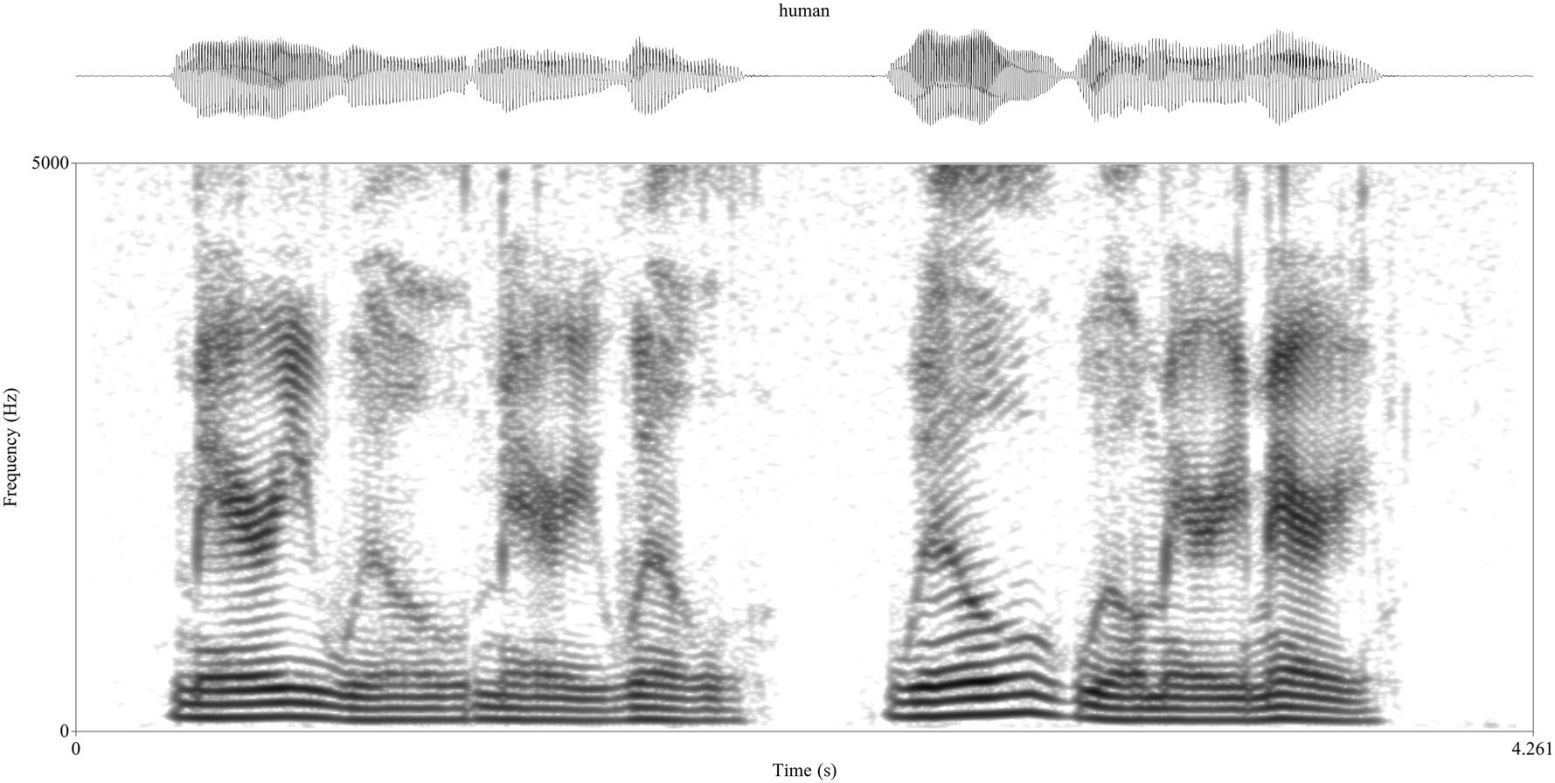
Human vocalization. The linguistic units in a human vocalization are not clearly discernible from the acoustic structure. Here, there are two sentences: “Mary will marry Will.” and “Will will marry Mary.” The human vocalization is a male speaker of American English: a dialect where “marry” and “Mary” are pronounced the same way. Spectrogram created in Praat (Boersma and Weenick 2022). Frequency range is 0–5 kHz. The spectrogram window length is 0.05 s

Take the acoustic signal in Fig. 2.

In their externalized form, unit boundaries in both spoken and signed language are not obvious. In Fig. 2, the only clear boundaries related to silence which would suggest two units. While there are similarities in the acoustic structure, the two utterances are clearly distinct (assuming the alien knows that formant structure is relevant to humans). Of course, the alien researchers could use more subtle acoustic cues—like transitions in formants, amplitude, or frequency—to find repeating patterns which will help to discover units or subunits. In this simple example, acoustic modulation within the two utterances suggest that they are both composed of units. Using changes in the second formant frequency (*F*2), the aliens could define two unit types (allowing for some within-class variation, of course) across the two utterances. One unit is associated with a lower *F*2 that rises then falls, the second has a higher *F*2 with a rise-fall-rise shape. Using conventions commonly found in birdsong analyses where different letters represent different unit classes, utterance one would have a structure of ABAB and utterance two is BBAA. This is, of course, not an accurate representation of the meaning of the two sentences. But, from an outsider perspective, it could take significant amounts of research to uncover the fact that human phrases can contain words that are acoustically similar but that differ not just in meaning but in their part of speech: [me̞ɹi] (pronunciation)—Mary (noun)—marry (verb); [wɪ̈l] (pronunciation)—Will (noun)—will (verb). Even if the aliens get this far, why would they then think that human language is structured hierarchically? “Mary will marry Will” and “Will will marry Mary” can both be analyzed with sequential structure.

The example in Fig. 2 is obviously designed to make it tough on the alien linguists. They would have much more data to work with so it could be easier to discover the linguistic structure underlying human utterances. Still, more data might not be helpful. Human languages are diverse and there may be no syntactic or morphological rule that holds for every language (Evans and Levinson 2009). Even if aliens have an excellent understanding of the units and structure of a diverse sample of world languages, they still might not uncover any data that require hierarchical structure. In a corpus analysis of European languages, Karlsson (2007) found that no written language had more than three levels of center embedding and in spoken language there were almost never any more than a single level.

If hierarchical constructions (or constructions that require hierarchical organization) are rare in the externalized form of human language, is hierarchical structure really that important for human linguistic capabilities, for the evolution of language, or for the evolution of communication? And what chance do human researchers have to discover hierarchical structure in the external signals of animal communication? This, of course, assumes that scientists even have an accurate understanding of the units and function of the communicative signal in non-human communication.

**Fig. 3:**
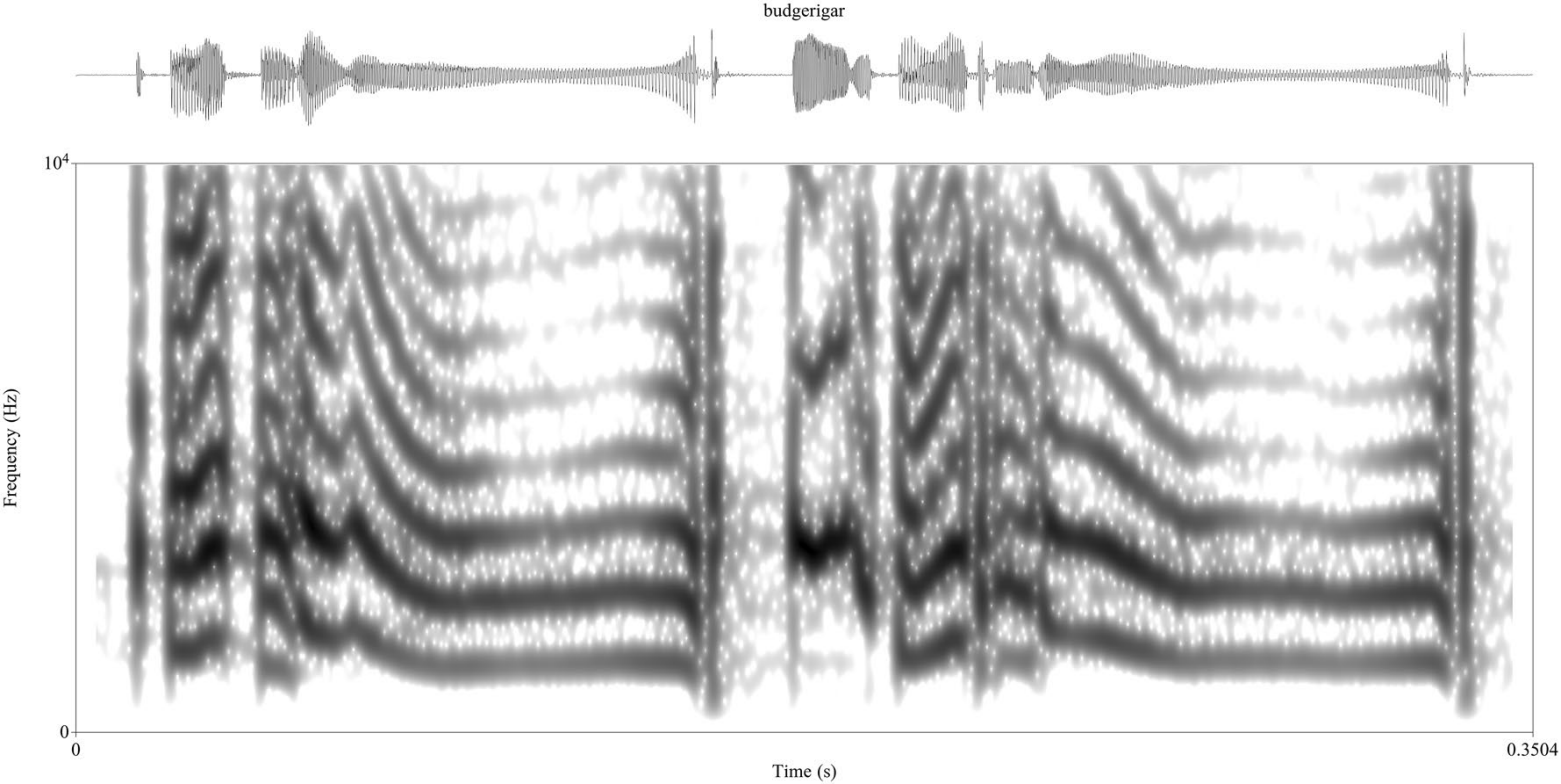
Budgerigar vocalization. Recording of a male that was housed at the University of Vienna (see Mann et al. 2021). Spectrogram created in Praat (Boersma and Weenick 2022). Frequency range is 0–10 kHz. The spectrogram window length is 0.005 s

Compare the human vocalization in Fig. 2 to an animal vocalization in Fig. 3. The signal in Fig. 3 is a section of a song produced by a male budgerigar. Songs usually consist of multiple unit types but can vary wildly from species to species in how many units are in a song and how stereotyped the ordering of the units is (Beecher and Brenowitz 2005). The number of units in a song depends on choices made by the researcher. For one, a “syllable” in animal song is commonly defined as a continuous trace on a spectrogram or a unit divided by small periods of silence; but the researcher must decide on the amount of silence that is needed to divide syllables. There are methods to help decide on a duration (Catchpole 1976; Isaac and Marler 1963) but these methods are better suited for species with more stereotyped song and are best used as a first approximation to be assessed with other external methods depending on the research question (like respiratory phases, Franz and Goller 2002). The repetition of acoustic structure can also help to decide on the number of units. This repetition also helps researchers define unit classes which are typically classified based on the similarities of their spectra-temporal properties.

Of course, even when using methods to help with objectivity, there is quite a bit of subjectivity in dividing a song. For the signal in Fig. 3, it is difficult to know how many units are present. Based on the size of the silent gaps, two complex units seems reasonable. But using even a small presence of silence, one could argue for roughly nine units. The long downsweep seems to repeat, as do some of the click/burst-like sounds. There also seems to be two other short harmonic sounds that repeat; one with a slight frequency increase, the other with a slight frequency decrease (with the latter a period of silence separating it from the longer harmonic downsweep is not present). So, does the song have two units that are in different unit classes? Or are there closer to nine to eleven units with five or six unit classes? Even though there are acoustically similar units (like the long downsweeping harmonic stack) are these units perceived similarly? At one extreme, budgerigars might not have any perceptual grouping of units at all. At another extreme, maybe they are like humans in that they have perceptual grouping but the ordering and the relationship between the individual sounds is more relevant than acoustic similarity of the units. As with the human example, relying on acoustics alone likely cannot solve the dilemma. A preliminary model can help researchers make better sense of the structure, but one cannot have too much faith in that model.

Ultimately, it is not obvious that comparing human language syntax with animal song "syntax" will be the most informative from a comparative standpoint. Among the uses humans make of language is sharing concepts. The function of song varies depending on the species, but the most empirically well-supported functions of song include mate attraction, territorial defense, and species recognition (Williams 2004). However, from the outside the function of human language may be seen similarly to animal song: it may appear to be used for social coordination (much like animal “calls”, which, just like songs, can be complex and learned though it is less likely than song, see Tyack 2020; Hughes et al. 1998) and mate attraction (much like animal “songs”).

This is not to say that comparative research cannot say anything about human syntax or hierarchical organization in animals. Far from it. If one is interested in finding parallels to human language syntax, one might focus on cognitive abilities, in addition to communication (or perhaps instead of, Fitch 2019). Seyfarth and Cheney (2017) postulate that the relationship between calls and social concepts in baboons requires complex, rule-governed computation which might serve as a precursor to language. To understand a call, a baboon has to understand what the call type is, which individual is calling, and—because the ranks of individuals are always changing—the social status of the vocalizing individual. Also, there is no strong evidence that human language syntax is actually “autonomous” (that syntax is separate from other domains of cognition as argued by Chomsky 1957, pp. 13–17) and there are many models of language where rules and regularities in syntax are emergent from meaning, social function and context, historical processes, and domain-general cognitive abilities (Croft 1995). In this sense, the amount of comparative research where syntax is relevant could actually be much larger; for instance, the relationship between call and meaning, "meaningful" call combinations, animal concepts, the organization of thought and action, information sharing, etc. might be informed by and inform on human language syntax.

Human language syntax serves as a poor model for animal vocal communication signals and vice versa. However, other domains of human language are more directly comparable to animal vocal behavior. There is a renewed interest in exploring the relationship between animal vocal behavior and traits related to human phonology (and related fields like phonetics, phonotactics, and prosody). In spite of important differences, there are widespread similarities in the mechanisms of vocal production and sound perception across species (Beckers 2011; Mol et al. 2017; Tierney et al. 2011). In fact, the emotional part of speech sounds appears to be shared among vertebrates (Filippi 2016; Filippi et al. 2017).

As mentioned earlier, silence is the most used cue used to define unit boundaries. Silence is a clear and (relatively) easy to define acoustic cue so it is, of course, incredibly helpful in understanding vocal behavior. However, for many species it is likely to be insufficient to capture the full complexity of their vocal system (see Fig. 3). If, for instance, an alien researcher tasked with analyzing human vocal behavior only used silence to divide the human acoustic signal, they might assume a much smaller inventory of sounds. Humans might have a few calls which are pretty stereotyped (at least within an individual) and more easily categorized by behavioral context such as alarm calls (e.g., cries, screams), threat displays (e.g., screams, yelling), and affiliative calls (e.g., laughter). Humans would then have another category of unstereotyped vocalizations (spoken language) which are used in a variety of behavioral contexts and are not easy to define or cluster. Perhaps these vocalizations are used in courtship—they do occasionally lead to copulation, after all. Of course, because humans can self-evaluate, researchers know that much of the interesting data in human speech occurs in vocalizations not divided by silent intervals. Segments, units divided by rapid acoustic transitions, form the basis for much of the research in phonetics and phonology.

To date, potential units within animal syllables—like those that exist in human phrases—have received very little attention. Jansen et al. (2013) describe calls composed of two segments in banded mongooses (*Mungos mungo*). In these “close calls”, a noisy segment is followed by a harmonic segment. The initial segment seems to serve as a cue to individual identity while the harmonic segment varies by behavioral context. Campbell’s monkeys (Ouattara et al. 2009) and dingoes (Deaúx et al. 2016) appear to have similar segmental systems where syllables (again, a “syllable” in non-human vocal communication is commonly defined as a unit divided by small amounts of silence or, similarly, a continuous trace on a spectrogram) that exist independently are combined to create novel multi-segment syllables.

These species are non-vocal learners and have relatively simple systems. Because they are simpler, segments are easier to identify. Segments are likely to exist in more complex systems but have not been described because even defining the syllable inventory is a difficult task. In birdsong research, units below the syllable are often referenced, but rarely analyzed (Williams 2004 refers to these units as “elements”). In the whistle-like sounds of cetaceans, frequency changes have been analyzed, though mainly as a measure of complexity in the continuous signal (May-Collado and Wartzok 2008; Tervo et al. 2011; Kershenbaum et al. 2013). May-Collado and Wartzok (2008) used the sum of frequency changes, or “inflection points”, in their analysis of bottlenose dolphin whistles. These metrics have been applied in non-cetaceans with continuous vocal signals, like the non-vocal learning wolves (Kershenbaum et al. 2017). Kershenbaum et al. (2017) and Kershenbaum et al. (2013) converted the continuous signals of wolves and dolphins, respectively, into sequences of segments using a Parsons algorithm. In this process, each sound was divided into a fixed number of segments. The segments were assigned to one of seven categories based on the magnitude and direction of the frequency change (e.g., large rise, small drop, flat, etc.). Because each signal ends up being a sequence of elements which are drawn from a finite set of element states, the authors could use the common metric of complexity, Shannon entropy. In a likely vocal learning bat species, the intermediate roundleaf bat (*Hipposideros larvatus*), Chi et al. (2020) traced the development of calls from just after birth to adulthood. They found that the adult syllables were created by the merging of syllables that—at early stages of development—had been independent. These data suggest that these calls are multi-unit but that the individuals throughout development gain greater degrees of motor control which allows them to produce tightly coordinated transitions between the units. Without tracing the development of these calls, the potential segmental system would not be obvious.

Looking at spoken human languages, the possible sound combinations that can be produced are innumerable. The number of sounds that combine to produce the theoretically infinite repertoire can be (more or less) listed in a few pages (e.g., the International Phonetic Alphabet fits a list of spoken language sounds and the subtle modifications to those sounds on a single page). For species with large or undefined syllable repertoire sizes, segmental approaches could help better define the systems and better understand how other species can build seemingly infinite systems. Budgerigars, for instance, are a prime candidate for a segmental approach. The most basic unit described until recently was the syllable, despite the fact that their syllables are non-stereotyped and non-repeating (Farabaugh et al. 1992). Therefore, Mann et al. (2021) applied a segmentation model to the non-stereotyped budgerigar syllables and found that budgerigars, like humans, had two broad classes of sounds: plosive sounds which were acoustically similar to the plosive class of human speech consonants (sounds like *p*, *t*, *d*, *g*) and harmonic sounds which were somewhat analogous to vowel-like sounds (e.g., *a*, *i*, *o*). Moreover, budgerigars preferentially start vocalizations with a plosive, while periodic signals are found within a syllable, much like with the human “CV preference”, the tendency for human languages to prefer burst-periodic patterns at the start of a vocalization (Fougeron and Keating 1997; Hyman 2008). There is no agreed upon explanation for this pattern in human languages, but human-specific hypotheses have been proposed (Prince and Smolensky 2004). While the similarities between budgerigars and humans could be random convergence, if we treat humans just like any other species, these data give us even less reason to appeal to human-specific mechanisms. Without segmental data, it is not possible to draw appropriate comparisons between animal communication and human language, and a critical tool in deciphering the meaning, function, and evolution of animal acoustic communication is lacking. Studying segmental data in non-human animals based on ideas from human language also may provide important insight into animal communication systems as a whole that would not be possible to investigate without the insider information obtained from being a member of the human species.

## Conclusions

Especially in more applied areas of research, such as medical research, other animals may be used as a model for humans. Here we showed how the opposite can also be true: using humans as a model for other species can lead to insight into the abilities of other animals. It has long been agreed that to understand animal cognition humans should not be considered as belonging to a separate category. As Darwin (1871, pp. 105) put it over 150 years ago, humans are different in degree and not in kind. The insights provided by experience with being a member of the human species can be very informative, but they can also lead one astray if the insights are not verified using a comparative approach. In other words, one cannot depend on intuitions of ones own species, but one also should not throw the baby out with the bath water. Intuitions as a member of the human species are an excellent starting point, but not an ending point, to self understanding.

**Fig. 4:**
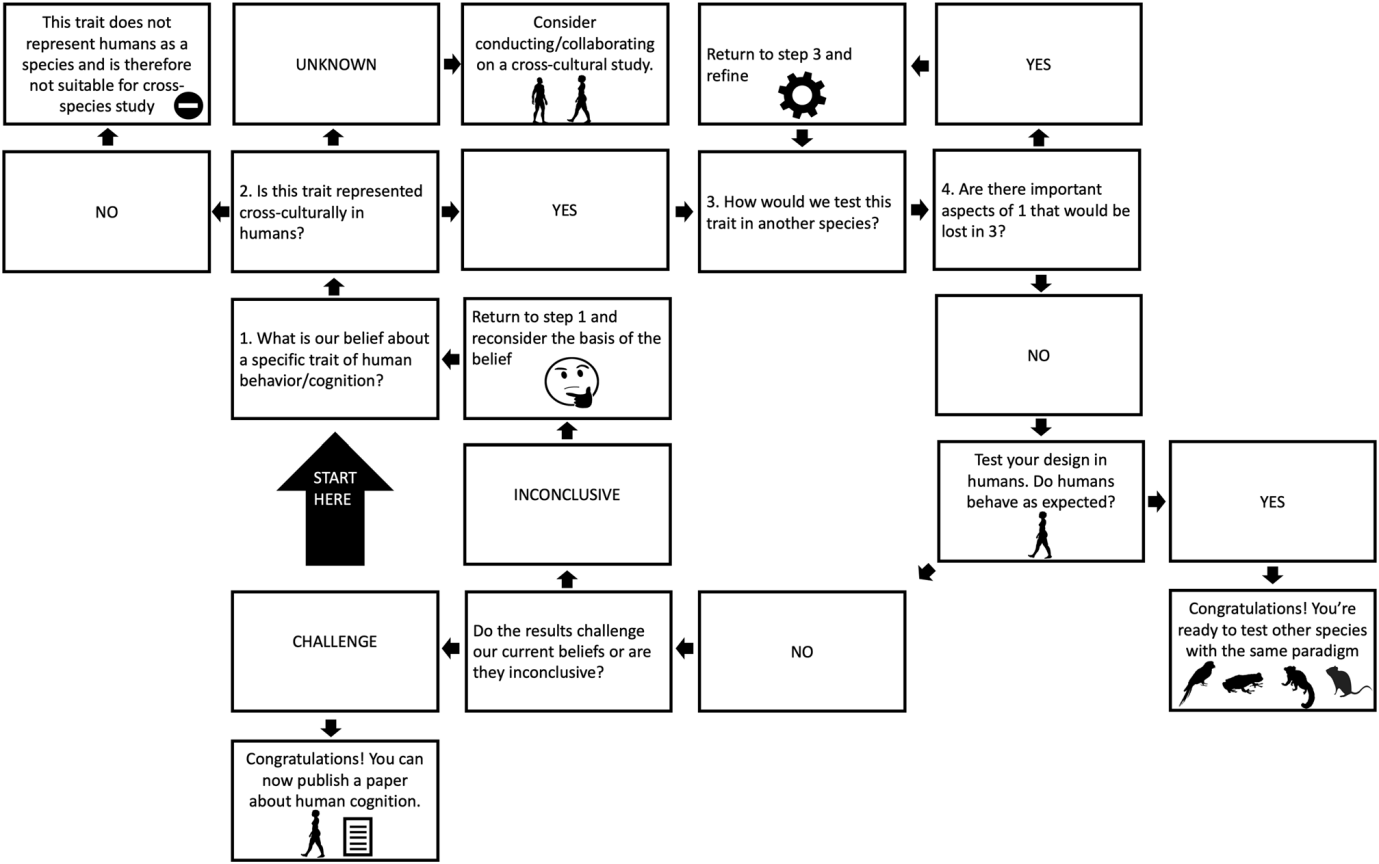
A flowchart showing how an objective human-centric approach to animal cognition can be applied

As we hope our examples here have demonstrated, to follow an objective human-centric approach takes more than simply applying insider knowledge about humans to animals. Here we have put together a flowchart illustrating how one can practically implement the three-step objective human-centric approach described in the introduction (see Fig. 4), effectively breaking down what things one needs to consider when moving from insider knowledge to an alien-researcher perspective (as described in Fig. 1). We hope that this approach will be more commonly applied in other areas of animal cognition.

Of course, the approach we present here is just one of many approaches to animal cognition and should not be used exclusively. If it were, a lot of interesting phenomena in species with traits that are outside of the scope of human behavior and cognition would remain unstudied. That would be disastrous, as we would not have a comprehensive understanding of cognition as a whole. Indeed, our objective human-centric approach can only lead to a deeper understanding of cognitive traits that are relevant to humans.

We fully agree with other authors who have argued that often we attribute intelligence to other species simply because they are doing something human-like, and not because of an objective measurement of intelligence (e.g., Bräuer et al. 2020). By focusing on human traits in our approach, it is important to note that one should not in any way assume that humans are the “gold standard” for any particular trait. In fact, if this process is done correctly, more often than not one should be able to find examples of species that exceed human abilities in one way or another (e.g., insects that synchronize more accurately to rhythms as discussed in the rhythm section). As a whole, an objective human-centric approach is not meant to place undue importance on the human species, instead it is an attempt to take the best parts of being a member of the animal kingdom without the anthropomorphic baggage that was prevalent in earlier animal cognition research. Now that the field has reached a point where the importance of considering the ecological relevance of particular tasks and questions to particular species is recognized, we believe it is now possible to more safely apply our approaches to humans and answer long sought-after questions about humans as a member of the animal kingdom while unlocking a deeper perspective of cognition as a whole.

## Data Availability

Not applicable.
